# Patient individual phase gating for stereotactic radiation therapy of early stage non-small cell lung cancer (NSCLC)

**DOI:** 10.1038/s41598-021-85031-w

**Published:** 2021-03-12

**Authors:** K. M. Kraus, M. Oechsner, J. J. Wilkens, K. A. Kessel, S. Münch, S. E. Combs

**Affiliations:** 1 School of Medicine and Klinikum Rechts Der Isar, Department of Radiation Oncology, Technichal University of Munich (TUM), Munich, Germany; 2 Institute of Radiation Medicine (IRM), Department of Radiation Sciences (DRS), Helmholtz Zentrum München (HMGU), Neuherberg, Germany; 3 Deutsches Konsortium für Translationale Krebsforschung (DKTK), Partner Site Munich, Munich, Germany

**Keywords:** Lung cancer, Radiotherapy, Applied physics

## Abstract

Stereotactic body radiotherapy (SBRT) applies high doses and requires advanced techniques to spare surrounding tissue in the presence of organ motion. In this work patient individual phase gating is investigated. We studied peripheral and central primary lung tumors. The internal target volume (ITV) was defined including different numbers of phases picked from a 4D Computed tomography (CT) defining the gating window (gw). Planning target volume (PTV) reductions depending on the gw were analyzed. A treatment plan was calculated on a reference phase CT (rCT) and the dose for each breathing phase was calculated and accumulated on the rCT. We compared the dosimetric results with the dose calculated when all breathing phases were included for ITV definition. GWs including 1 to 10 breathing phases were analyzed. We found PTV reductions up to 38.4%. The mean reduction of the lung volume receiving 20 Gy due to gating was found to be 25.7% for peripheral tumors and 16.7% for central tumors. Gating considerably reduced esophageal doses. However, we found that simple reduction of the gw does not necessarily influence the dose in a clinically relevant range. Thus, we suggest a patient individual definition of the breathing phases included within the gw.

## Introduction

Delivery of extracranial ablative doses of ionizing radiation in one or a few treatment fractions is commonly known as stereotactic body radiotherapy (SBRT)^1^. The development of sophisticated treatment and imaging techniques, such as image-guided radiation therapy (IGRT), improved the precision of dose delivery. Thus, dose escalation becomes safely feasible. SBRT offers the possibility to apply ablative doses to the tumor volume while sparing healthy surrounding tissues. SBRT is the current standard of care for inoperable early stage Non-small cell lung cancer (NSCLC)^2–6^. Especially patients with pulmonary or cardiological comorbidities precluding surgery are clearly benefitting from the non-invasive nature of the SBRT procedure. Previous studies revealed excellent local control rates of 97%^7^ for peripherally located inoperable early stage NSCLC. Usually, SBRT of peripheral lesions is related with a small risk of adverse events^8^. When treating centrally located tumors the risk for the occurrence of adverse events is increased due to the proximity of bronchial and mediastinal structures^9^. However, several studies revealed the safety of SBRT for central lesions^10,11^. Further reduction of toxicities could be achieved by a reduced dose per fraction leading to a biologically effective dose (BED) below 100 Gy together with an increased risk of local failure^12^.

Organ motion due to breathing represents another challenge for safe application of high doses with steep dose gradients in close proximity to organs at risk (OAR). Misdosage of a single dose can in case of SBRT magnify the dose to normal tissue compared to fractionated radiotherapy. The most common approach to overcome motion induced misdosage is the ITV (Internal Target Volume) concept by which an additional margin is defined encompassing the tumor in all respiratory motion phases of a 4D CT (Computed Tomography)^13^. Further motion management strategies have been developed such as tracking of the tumor or gating, which means to pause the irradiation when the tumor moves out of the predefined motion window^14^. Several studies compared SBRT lung cancer treatment with motion management strategies to an ITV approach and found similar efficacy^15,16^. Lately, Heard et al.^17^ investigated treatment volume reduction by a decreased number of breathing phases for ITV definition and the resulting dose coverages and found underdosage of the PTV up to 12.3%.

However, SBRT using ablative doses for lung tumors close to critical OAR is still used cautiously due to the increased risk of severe side effects. Gating has been used long before, however, usually only by applying an arbitrarily defined gw aiming to deliver a highly conformal dose on the costs of an increased treatment time.

This paper aims to improve SBRT of lung tumors in close proximity of OAR in the presence of motion. We quantify PTV reduction by breathing phase gating and analyze the resulting dosimetric consequences for stereotactic treatment of lung tumors. We simulate gating by dose accumulation over the used breathing phases and focus on a patient individual gating approach.

## Methods and material

### Data

We investigated four peripherally and three centrally located lung tumors. All patients suffered from early stage lung cancer (T1a to T2a, 8th edition of TNM staging). Locations differed and are summarized in Table 1. Two peripheral lesions were located in the right lower lung (RLL) and two were located in the left upper lung. Two central lesions were found in the left hilar lung and one in the right lung. Patient ages ranged from 63 to 83 years.

**Table 1:** Patient data

Patient case	Tumor location	Patient age	TNM
1	RLL	81	cT1c cN0 M0
2	LUL	72	cT1 cN0 cM0
3	LUL	79	cT1 cN0 cM0
4	RLL	83	cT1b cN0 M0
5	RL hilar	74	cT1b2 cN0 M0
6	LL hilar	63	cT1c cN0 cM0
7	LL hilar	80	pT2a pN0 cM0

*RLL* right lower lung, *LUL* left upper lung, *RL* right lung, *LL* left lung.

### Motion management simulation

**Figure 1:**
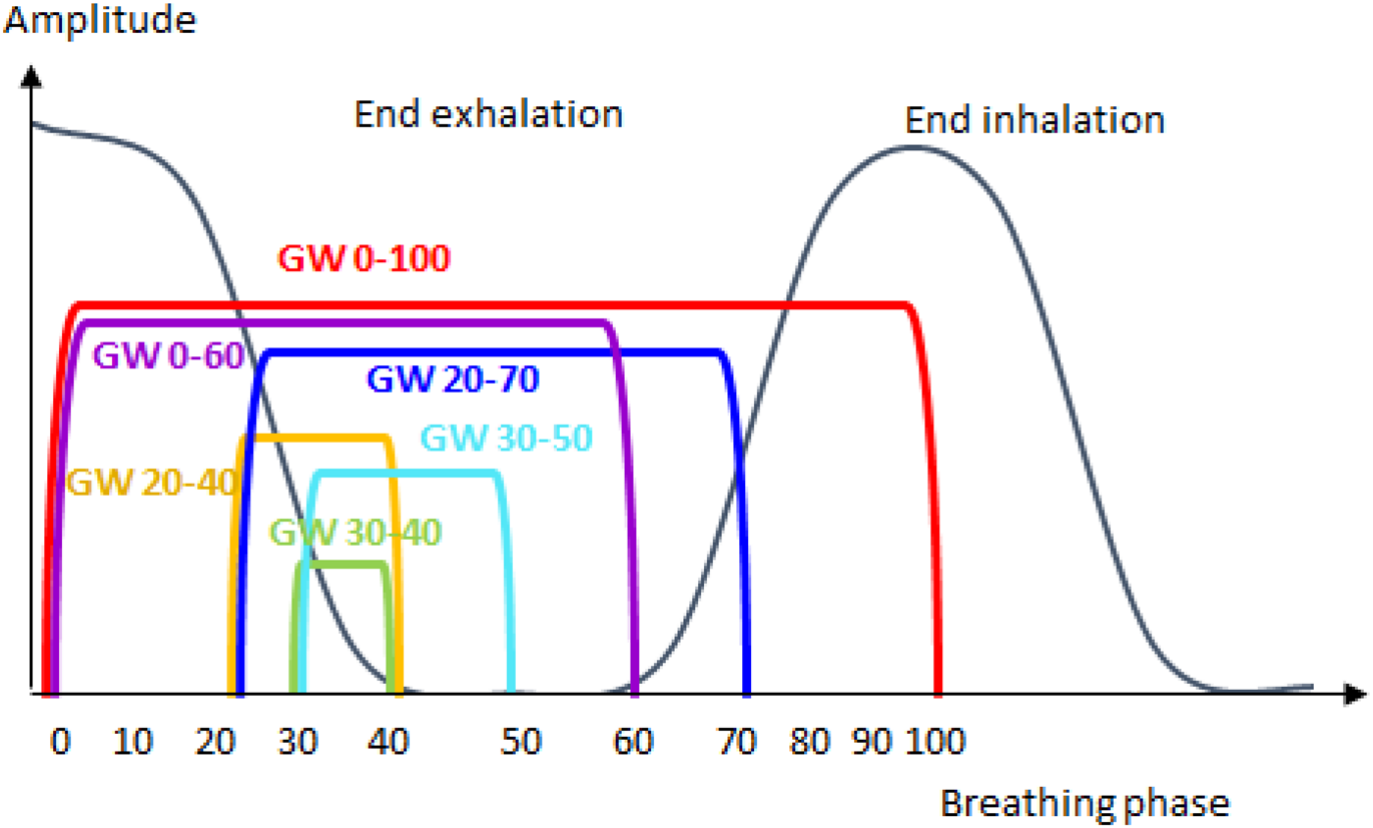
Schematic drawing of a breathing cycle. Breathing phases are defined from 0 (end inspiration) to 50 (end exhalation) and to 100 (end inhalation). The different gating windows (gw) investigated in this study are depicted.

For all patients, a 4DCT was acquired on a Somatom Emotion 16 CT (Siemens Healthineers, Erlangen, Germany) comprising ten respiratory phases, where phases are indicated in percent of the breathing cycle. The originally acquired breathing phases were manually adapted so that 0 is assigned to maximum inhalation and 50 is assigned to maximum exhalation as depicted in Fig. 1. We delineated the gross tumor volume (GTV) on the slow planning CT (pCT) acquired during free respiration. Tumor delineation was performed manually for all CT phases. An ITV was defined comprising the GTV in all ten phases of the 4DCT for the standard treatment plan and a limited number of phase CTs for gating. An additional isotropic margin of 5 mm was added to the ITV resulting in the PTV. For comparison to standard treatment procedure, one volumetric modulated arc therapy (VMAT) treatment plan with typically two to three arcs was calculated based on the planning CT using Eclipse 15.6 treatment planning software (Varian Medical Systems, Palo Alto, CA, USA).

A dose of 45 Gy delivered in 3 fractions was prescribed to the 65% isodose. 95% of the prescription dose should cover 98% or 99% of the PTV. Dose constraints were assigned according to^5^. For simulation of gating, different gws comprising a limited number of respiratory phases were assigned and are depicted in Fig. 1. The gw size varies from 10% for gw 30, when only one phase CT is used for dose calculation to 100% when all breathing phases are used. The choice of gws is based on the individual tumor location and size as well as tumor motion. Patient case 4 and case 7 where chosen as representatives for extreme situations. For patient case 4 with a peripheral tumor location and the largest amount of tumor motion, we chose three different gws, that were visually chosen and represent different interesting scenarios. Gw 30 was chosen as an extreme example using a single breathing phase for gating simulation. Gw 20–40 was chosen as a potentially practically applicable gw where the tumor motion extend is comparably small. Gw 0–60 was chosen for patient case 4 as a scenario comprising the major tumor motion, however, with a reduced number of breathing phases compared to the conventional ITV concept using all phases. Patient case 7 represents a central tumor close to critical organs. Here, we chose gw sizes covering one (gw 30), two (gw 30–40), three (gw 30–50) and six (gw 20–70) breathing motion phases. There is a focus on small gw sizes due to the critical tumor location. The particular phases where chosen with respect to the motion of the tumor and OAR.

Treatment plan parameters from the standard treatment plan were copied to a single phase CT that was closest to maximum inhalation called rCT. The standard treatment plan was re-optimized and the dose was re-calculated on the rCT and called the base plan. This base plan was then copied and re-calculated without optimization on the residual respiratory phases defined for the investigated gws resulting in the phase plans. For example, for gw 30–50, the standard treatment plan from the pCT was copied to phase 30 and re-optimized on the phase CT of breathing phase 30 and called base plan. This plan was copied to phases 40 and 50 and the dose was re-calculated without optimization. The doses from these phase plans were mapped to the rCT by deformable image registration using the open-source image registration framework Plastimatch (www.plastimatch.org). B-Spline multi-stage deformable image registration was derived between the phase CTs and the rCT. The resulting transformation was applied to the phase dose distributions and accumulated on the rCT to calculate the sum dose that was gathered over the used respiratory phases. The dose volume histogram (DVH) was calculated on the rCT for gating and on the pCT for the standard treatment plan. The dose to 2%, 50%, 95% and 98% of the PTV was analyzed. For OAR, the maximum and mean doses were derived. For the lung and the esophagus, we calculated the volume receiving 20 Gy and 17.7 Gy. Additionally, the PTV reduction is calculated compared to the PTV based on the ITV comprising all phases. Tumor motion was analyzed using the center of mass motion in cranio-caudal (cc) and left–right (lr) direction. Results are provided in Table 2.

**Table 2:** Motion, PTV sizes, PTV reduction and dose parameters for peripheral and central tumor.

Case number and case location peripheral	GW (phase)	PTV (cm^3^)	PTV reduction		Max GTV motion (mm)	PTV			V_20Gy_ [lung] (%)	V_20Gy_ [lung] red. (%)	D_mean_ [lung] (Gy)	D_max_ [esophagus] (Gy)	V_17.7 Gy_ [esophagus] (cm^3^)	D_max_ [heart] (Gy)	D_mean_ [heart] (Gy)
			(%)	(cm^3^)		D_2%_ (Gy)	D_50%_ (Gy)	D_95%_ (Gy)							
1 RLL	30–50	44.7	24.9	14.8	9.8. cc	66.1	54.2	46.7	7.5	11.8	6.6	7.9	0.0	15.6	2.4
	0–100	59.5	–	–		67.0	55.0	47.7	8.5		7.0	10.7	0.0	13.9	2.7
2 LUL	30–50	31.7	19.9	7.9	4.6. cc	68.4	55.6	42.8	5.5	3.5	4.4	14.2	0.0	13.3	2.9
	0–100	39.5	–	–		70.3	56.1	45.3	5.7		4.6	15.7	0.0	13.2	3.2
3 LUL	30–50	22.1	35.5	12.2	5.8. lr	58.8	50.6	40.5	4.6	33.8	4.1	7.8	0.0	6.3	0.3
	0–100	34.3				59.2	50.5	39.0	7.1		5.5	5.8	0.0	5.1	0.2
4 RLL	30	40.7	38.4	39.1	17.8. cc	71.1	56.8	45.6	6.0	68.3	5.7	8.9	0.0	13.1	3.9
	20–40	61	18.2	18.8		69.1	55.8	42.5	8.0	20.8	7.1	8.3	0.0	12.3	4.8
	0–60	69.6	9.9	10.2		70.6	52.8	35.2	8.5	15.8	7.4	8.0	0.0	13.5	4.8
	0–100	79.8	–	–		69.6	56.4	42.9	10.1		8.2	8.6	0.0	14.3	5.4
Mean									7.2	25.7	6.1	9.6	0.0	12.1	3.0
Std									1.6	21.2	1.3	3.1	0.0	3.3	1.7

Case number and case location central	GW (phase)	PTV (cm^3^)	PTV reduction		Max GTV motion (mm)	PTV			V_20Gy_ [lung] (%)	V_20Gy_ [lung] red. (%)	D_mean_ [lung] (Gy)	D_max_ [esophagus] (Gy)	V_17.7 Gy_ [esophagus] (cm^3^)	D_max_ [heart] (Gy)	D_mean_ [heart] (Gy)
			(%)	(cm^3^)		D_2%_ (Gy)	D_50%_ (Gy)	D_95%_ (Gy)							
5 RL hilar	30–50	15.3	11.2	1.9	3.7. cc	69.4	57.4	46.6	1.8	70.9	2.8	29.3	2.4	25.0	1.0
	0–100	17.3	0	0	0	70.6	58.9	45.9	6.2		3.1	56.7	8.7	51.3	2.6
6 LL hilar	30–50	38.4	20.4	9.9	3.9. cc	77.8	63.7	49.3	14.4	3.1	7.4	13.3	0.0	6.7	0.4
	0–100	48.3	0	0	0	79.5	63.2	48.4	17.5		8.5	17.2	0.0	8.6	0.4
7 LL hilar	30	27.9	33.1	13.8	5.3. cc	65.8	56.7	45.9	16.0	6.3	9.8	43.0	5.3	33.3	2.1
	30–40	28.7	31.3	13	–	65.5	56.6	46.5	15.3	7.0	9.8	44.3	5.3	35.3	2.3
	30–50	32.7	25.4	10.6	–	66.1	56.6	46.2	16.2	6.1	10.2	46.8	5.6	35.2	2.4
	20–70	34.7	16.8	7	–	65.8	56.1	46.7	17.9	4.4	10.7	46.1	7.5	34.2	2.4
	0–100	41.8	0	0	0	67.6	55.5	46.3	22.9		12.1	46.5	6.1	36.6	2.5
Mean									14.2	16.8	8.3	38.1	4.5	29.6	1.8
Std									6.4	26.6	3.3	14.8	3.1	14.2	0.9

Lung volume receiving 20 Gy V_20Gy_ [lung] for peripherally and centrally located lung tumors are reported. Maximum dose for the esophagus (D_max_ [esophagus]) and V_17.7 Gy_ [esophagus] indicating the esophagus volume receiving 17.7 Gy are given. Maximum (D_max_ [heart]) and mean heart doses (D_mean_[heart]) are reported. Cc stands for cranio-caudal and lr for left–right direction, respectively.

### Ethics approval and consent to participate

The ethical committee of the Technical University of Munich has approved the retrospective study protocol. All patients gave their written informed consent for radiotherapy. All methods were performed in accordance with the relevant guidelines and regulations.

## Results

### PTV Reduction and tumor motion

**Figure 2:**
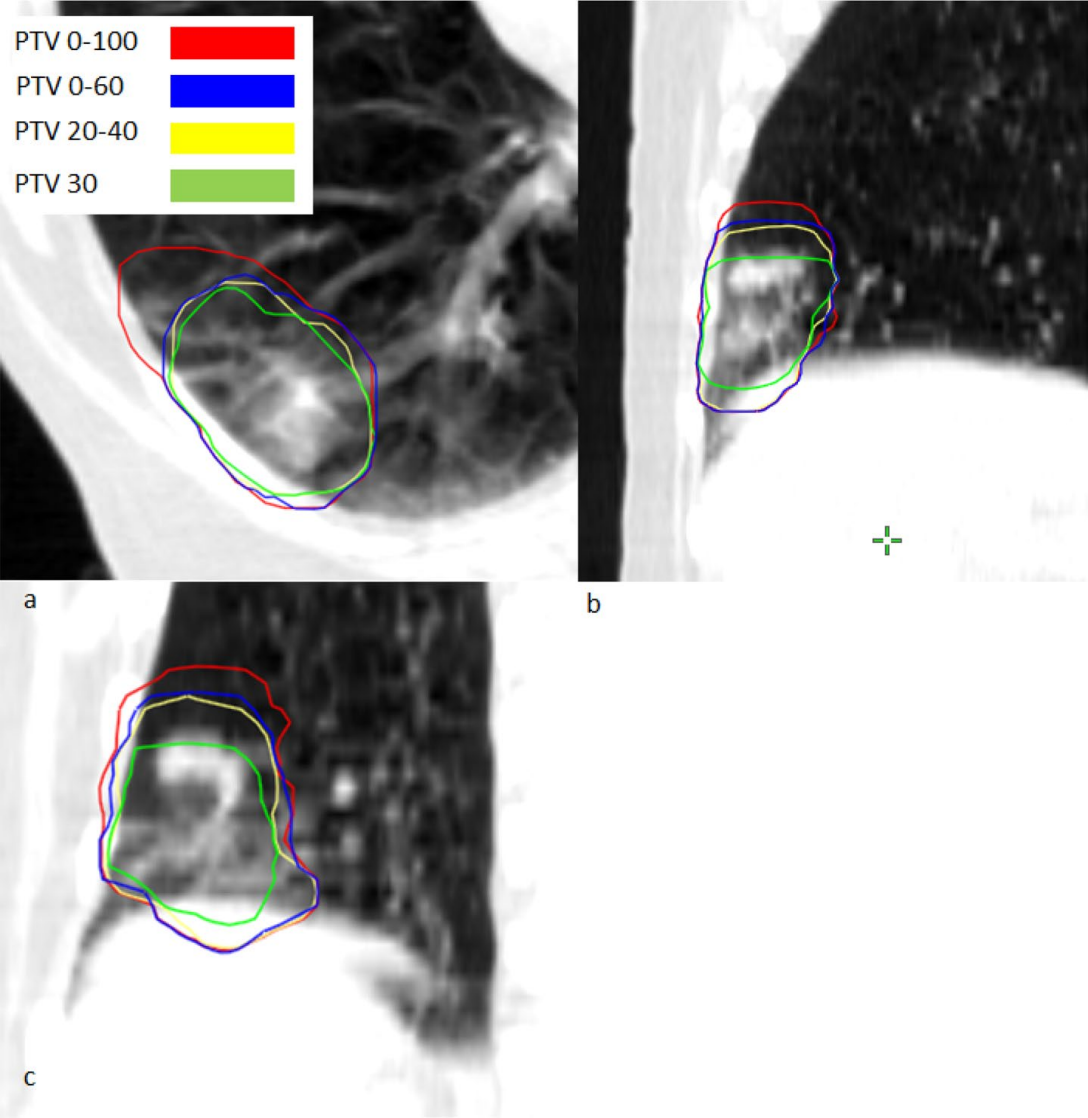
PTV contours for different gating window sizes for patient case 4 in CTs in transversal (**a**), sagittal (**b**) and coronal (**c**) view.

**Figure 3:**
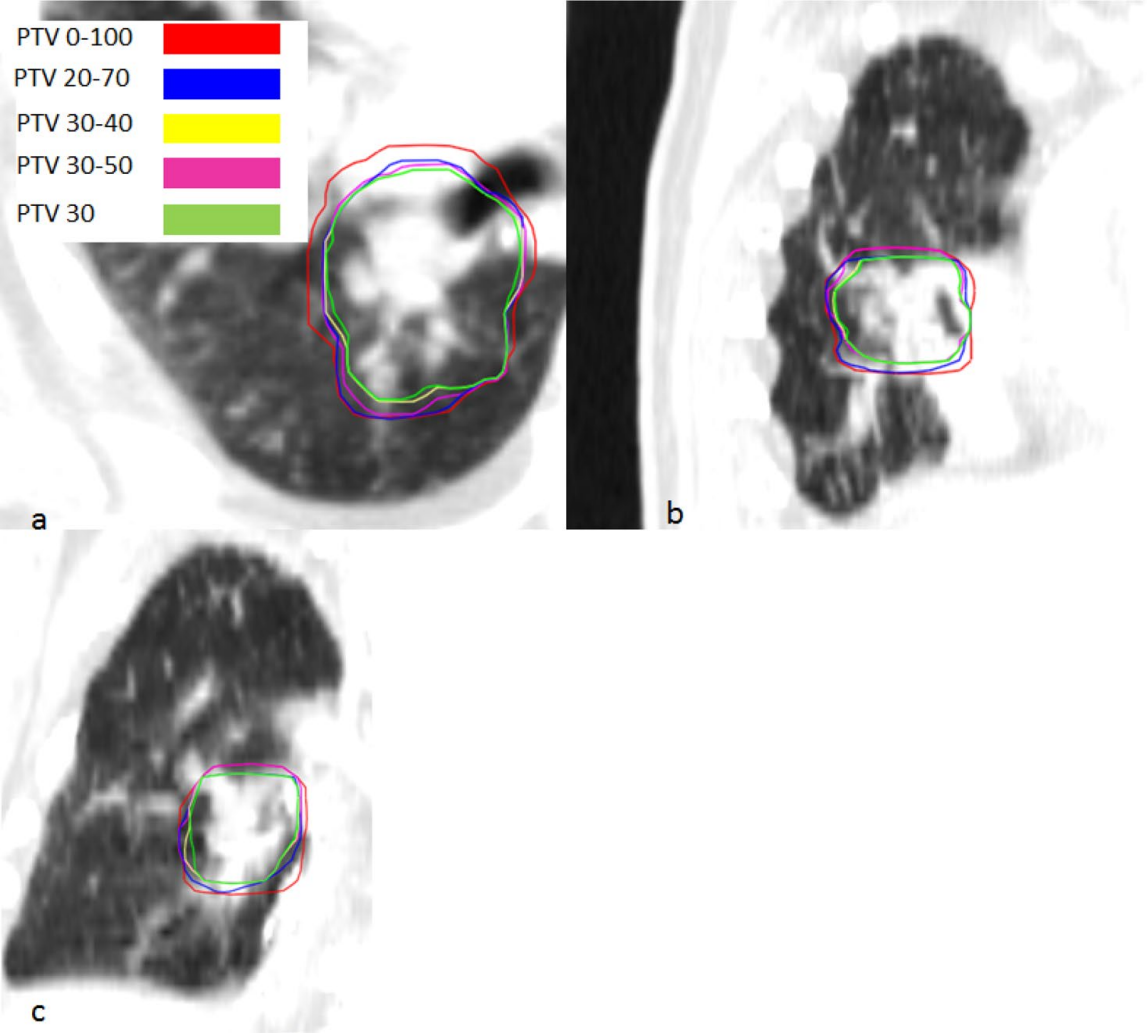
PTV contours for different gating window sizes for patient case 7 in CTs in transversal (**a**), sagittal (**b**) and coronal (**c**) view.

**Figure 4:**
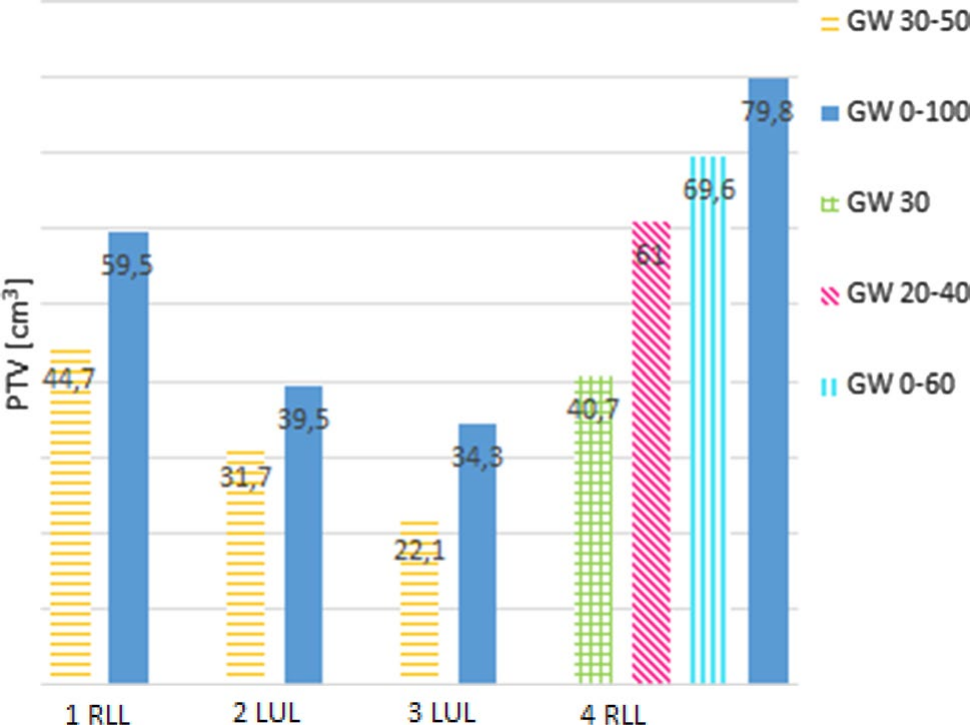
PTV in cm^3^ for peripheral tumors for different gating window sizes. The horizontal axis caption refers to the patient case and the location.

**Figure 5:**
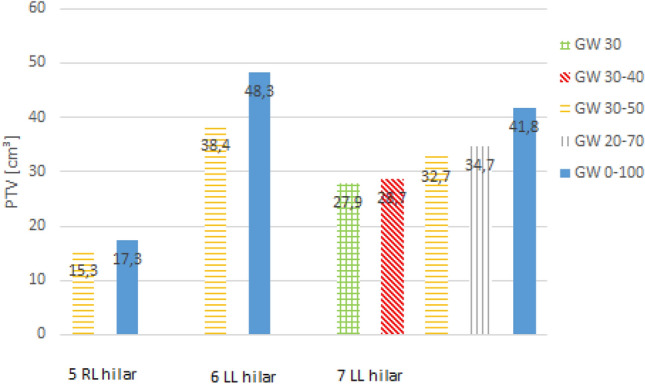
PTV in cm^3^ for central tumors for different gating window sizes. The horizontal axis caption refers to the patient case and the location.

An overview of PTV reduction, GTV motion and dose values for the PTV and OAR are given in Table 2. Maximum PTV reduction of 38% and 33% was observed for a peripheral tumor of patient case 4 and for a central tumor of patient case 7, respectively. The largest absolute reduction of the PTV was found for patient case 4 for which the PTV was reduced by 39.1 cm^3^ (38.4%). PTV reduction for patient cases 4 and 7 are shown in Figs. 2 and 3. Numeric PTV sizes for all patient cases are presented in Figs. 4 and 5. These were the tumors that showed the largest motion amplitudes. The tumor moved by 17.8 mm and 5.3 mm in cranio-caudal direction for patient case 4 and 7, respectively.

### Dose evaluation

**Figure 6:**
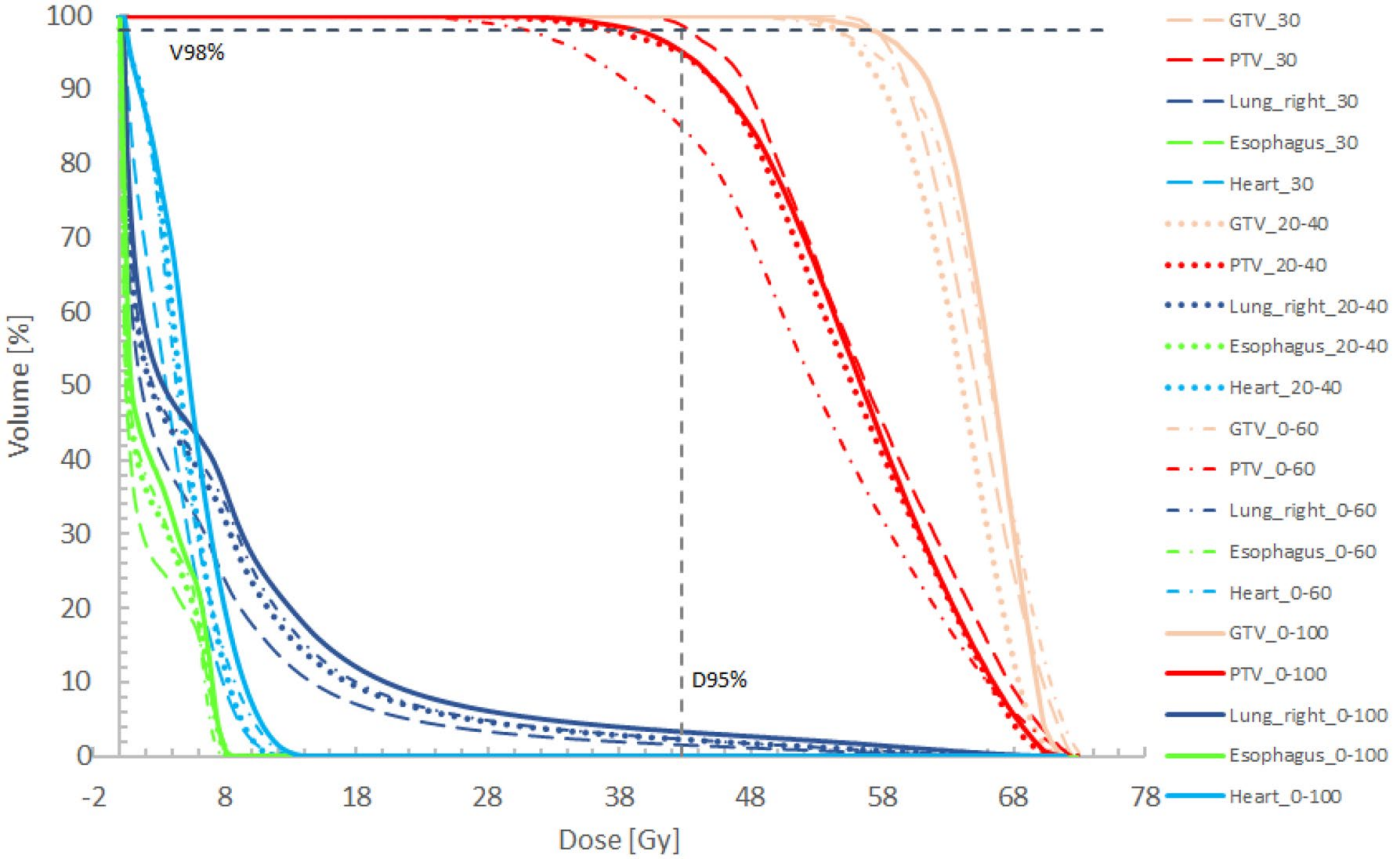
DVHs for patient case 4 for gating windows including phase 30 (dashed), phases 20–40 (dots), phases 0–60 (dashed-dots), and all phases (0–100) (bold solid) included within the gating window. A dashed vertical line showing 95% of the prescription dose as well as a dashed horizontal line indication 98% of the PTV are depicted.

**Figure 7:**
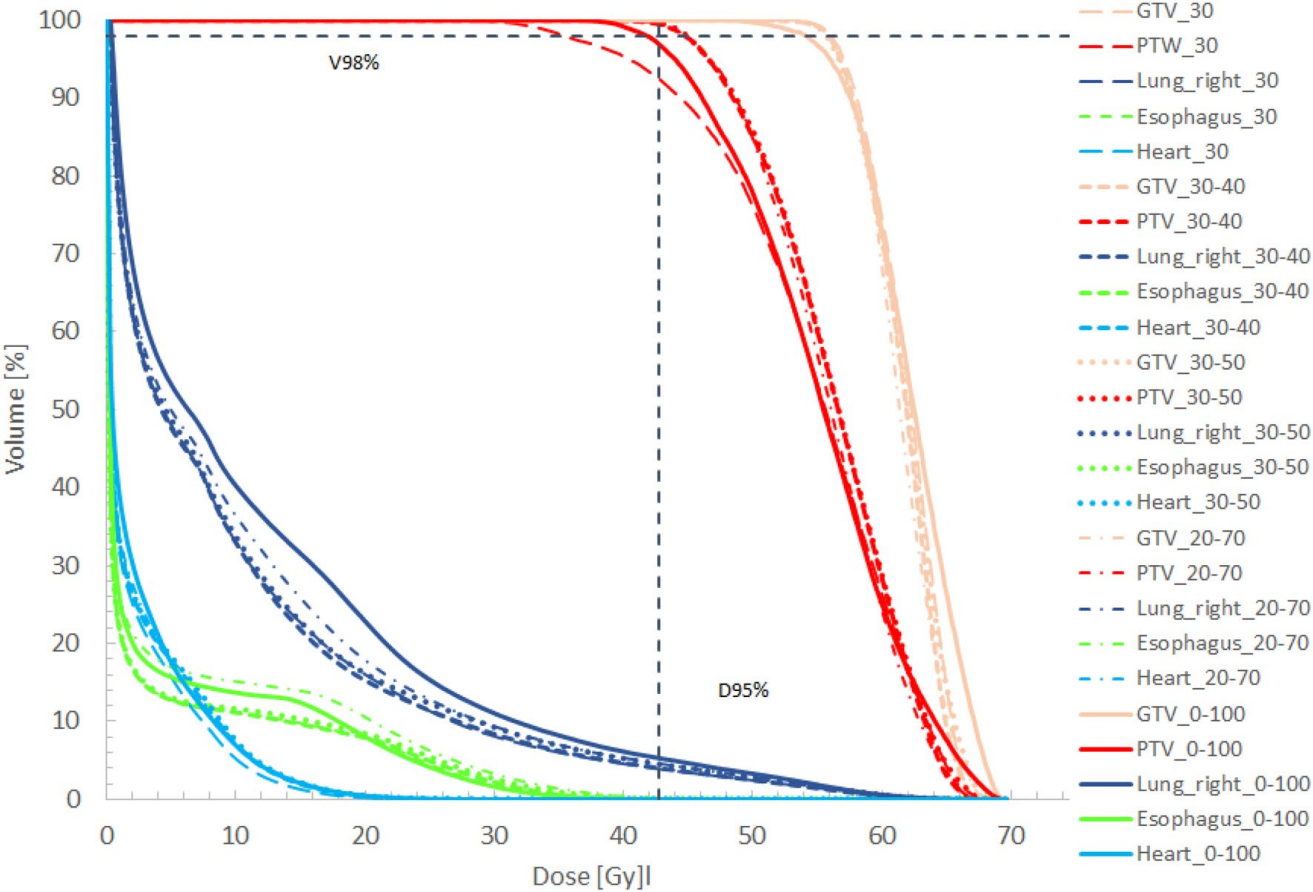
DVHs for patient case 7 for gating windows including phase 30 (dashed), phases 30–40 (bold, dashed), phases 30–50 (dots), phases 20–70 (dahed-dots) and all phases (0–100) (solid) included within the gating window. A dashed vertical line showing 95% of the prescription dose as well as a dashed horizontal line indication 98% of the PTV are depicted.

Dose coverages of the PTV are depicted in Figs. 6 and 7. The largest differences in the dose to 95% of the PTV were found for patient case 4. D_95%_ was reduced from 42.9 Gy when all ITV phases were included to 35.2 Gy when breathing phases from 0 to 60 were used for ITV definition. For central tumors, D_95%_ was not significantly influenced.

Analyzing the lung dose the volume receiving 20 Gy was less than 10% for almost all peripheral tumor cases. The smallest lung volume of 4.6% receiving 20 Gy was found for patient case 3 when a gw comprising breathing phases 30 to 50 was applied. Comparing this to V_20Gy_[lung] of 7.1% when all breathing phases were used a reduction by 2.7% could be observed. For peripheral tumors, we found a mean V_20Gy_ [lung] of 7.2% ± 1.6%. For central tumors, the dose to the lung varied. The mean V_20Gy_ [lung] was 14.2% ± 6.4%. Whereas for patient case 5 V_20Gy_ [lung] was 6.2% when all breathing phases were used for ITV definition and 1.8% for gating using breathing phases from 30 to 50. This can be explained by the small PTV of 17.3 cm^3^ and 15.3 cm^3^ using 10 breathing phases for ITV definition and using only breathing phases 30 to 50 for patient case 5. For patient case 7, a reduction of V_20Gy_ [lung] with reduction of the gw can be observed. Whereas V_20Gy_ was 22.9% for the conventional ITV concept. This value was reduced significantly when gating was applied. The smallest lung volume receiving 20 Gy was found when only two breathing phases were used for gating simulation. Further reduction to only one breathing phase used for gating did not further reduce V_20Gy_ for the lung. This can be explained by the differences caused by new treatment plan optimization that is performed for the plan based on the ITV only using breathing phase 30 and for the plan using an ITV based on breathing phases 30 and 40. This can lead to small differences in the dose of these base plans.

**Figure 8:**
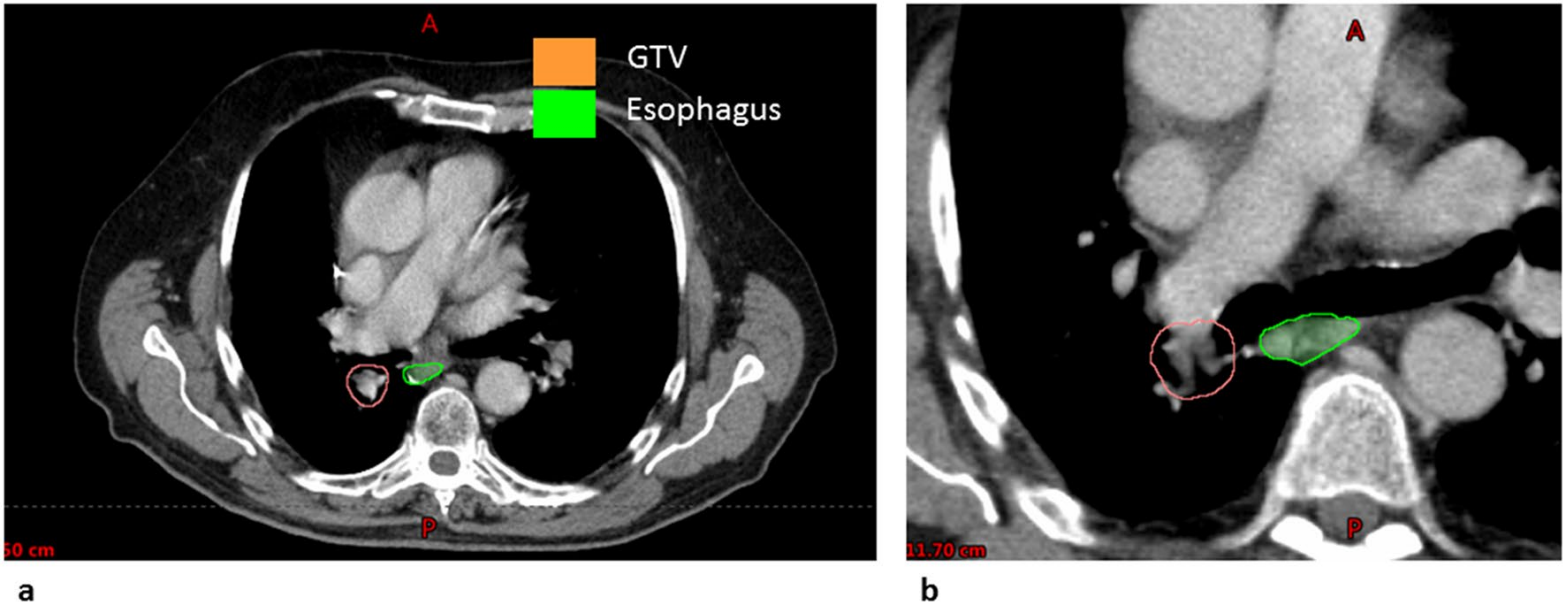
CT in transversal view (**a**) of patient case 7 showing the proximity of the GTV to the esophagus. Figure (**b**) shows a zoomed transversal view for the same patient but a different CT slice.

For peripheral tumors, the maximum esophageal doses could be kept below 16 Gy. The largest maximum dose of 15.7 Gy could be observed for patient case number 2 when all ITV phases were applied for ITV definition. This was reduced to 14.2 Gy when gating with a gw of three breathing phases was used. Obviously, for central tumors the maximum doses of the esophagus are much higher. This is due to the very close proximity of the PTV and esophagus as shown in Fig. 8 for patient case 7. Doses to the esophagus were small and thus not relevant regarding expected toxicities for peripheral tumor. For central tumors, esophageal volumes receiving 17.7 Gy were close to 0 for patient case 6. For patient case 5, V_17.7 Gy_ was 8.7 cm^3^ for the conventional ITV concept and could be remarkably reduced to 2.4 cm^3^ when a gw using three breathing phases was used. For patient 7, a general trend towards reduced esophageal doses for smaller gw sizes could be observed. The same applies to the heart. Interestingly, there was no large difference in esophageal D_max_ or V_17.7 Gy_ for a gw comprising two breathing phases compared to one breathing phase. When looking at maximum doses to the heart the largest reduction was found for patient case 5. For the conventional ITV concept using all breathing phases for ITV definition, the maximum dose to the heart was 51.3 Gy whereas for a gw of three phases the maximum heart dose was found to be 25 Gy.

## Discussion

SBRT is widely used for treatment of inoperable primary lung cancer and pulmonary metastases. Proximity to OAR such as the esophagus, the heart and vessels as well as the dose to healthy lung can hinder the application of high and ablative doses. Breathing motion adds another challenge to the problem of save delivery of high doses to the tumor. Therefore, reduction of the PTV as well as motion mitigation techniques can essentially contribute to overcome these challenges.

In this work we investigated the PTV reduction regarding tumor location and breathing motion amplitude. We found PTV reductions ranging from 9.9 to 38.4% for gw sizes including 1 to 7 breathing phases. Obviously, with fewer breathing phases included in the gw the PTV sizes were reduced. Maximum tumor motion was found up to 17.8 mm in cranio-caudal direction for peripheral tumors and up to 5.3 mm for central tumors. Recently, Heard et al.^17^ compared ITV and PTV sizes when all or a reduced number of breathing phases were used for tumor delineation and found underestimation of the ITV size up to 34.7%. Maximum motion amplitudes were 9.2 mm in cranio-caudal direction. The work of Heard et al., however, was not performed to explore gw sizes but rather for investigation of treatment efficacy by using fewer breathing phases for tumor volume delineation. Nevertheless, ITV size variation can be compared. In this work we could find similar PTV/ITV reduction using a subset of breathing phases for ITV definition.

The constraint of V_20Gy_ [lung] < 10% according to the ROSEL study^18^ could not be kept in all presented cases. For peripheral tumors, we found a mean V_20Gy_ [lung] of 7.2% ± 1.6% and 14.2% ± 6.4% for central tumors, respectively. For peripheral tumors, Ding et al.^19^ found a mean V_20Gy_ of the lung of 5.3% ± 3.6% using a dose prescription of 60 Gy to 95% of the tumor. In this work we revealed reduced doses to the ipsilateral lung with smaller gw sizes. The maximum reduction of V_20Gy_ of the lung was 68.3% for peripheral tumors and 70.9% for central tumors compared to the all phases ITV concept. The mean reduction of V_20Gy_ of the lung, however, was 25.7% for peripheral and 16.8% for central tumors. The PTV for the one scenario where we observed a V_20Gy_ reduction of the lung of 70.9% was very small (17.3 cm^3^) leading to this relatively large percentage reduction of volume. Prunaretty et al.^16^ found a reduction of V_20Gy_ [lung] by 17.5% for motion smaller than 1 cm and up to 33% for greater motion amplitudes compared to a conventional ITV approach using all breathing phases. Kim et al.^20^, however, found only small, clinically not relevant differences of V_20Gy_ [lung] when gating was applied. Comparability with the here presented data is limited due to a very different approach of gating simulation. Whereas Kim et al.^20^ chose the mid-exhalation phase for GTV definition and expanded it by 5 mm for motion simulation, we defined individual gws and accumulated the dose from each breathing phase. This simulation procedure represents the optimal approach to the real situation when dose is delivered in the presence of motion. Surely, the data presented in this work differs in certain aspects compared to the results found by others^16,17,20,21^ and clearly, the number of patient cases is small for general statements. However, we thoroughly picked patient cases that have the potential to reveal the remaining obstacles of lung SBRT and that can be addressed by the method presented and that also might be missed by the study of other patient cohorts.

We also revealed scenarios where further reduction of the number of breathing phases included within the gw did not lead to improved sparing of OAR. For the peripheral tumor case 4, the maximum dose to the esophagus was smallest when 7 breathing phases were used. This can be explained by the tumor location with respect to the esophagus in the different breathing motion phases and reveals a substantial aspect of this work. Interestingly, for the central tumor case 7 the maximum dose to the esophagus is below 45 Gy for a gating window size of 1 or 2 breathing phases whereas the dose is increased over 46 Gy for 3 or more breathing phases within the gw. This reveals, that for definition of the gw the number breathing phases included as well as the tumor position in the different motion phases should be taken into account. These findings represent the rational for patient individual phase gating for lung SBRT. However, one should not miss to consider the potential misdosage that might more likely be associated with very small gw sizes comprising only one or two breathing phases. Thus, application of very small gw sizes might increase potential dosimetric errors and careful gating performance including reliably reproducible breathing phases must be assured. Additionally, reduction of the gw size comes with the price of treatment time prolongation. This effect has been studied long before^22,23^. Together with dose fractionation, dose delivery might no longer be efficient and a reduced number of oncological patients can be treated. Therefore, very small gw window sizes might not be practical in the clinical routine due to its dosimetric sensitivity and due to treatment time prolongation.

Another aspect that should be considered is the binning of the breathing cycle. Usually, a 4DCT is binned into 10 equidistant discrete parts. It is assumed that the motion in between the chosen breathing phases is negligible. Obviously, this is a method grounded on an ideal, however, not always realistic situation. In our work, we revealed that individual gws can lead to better dosimetric results than just minimizing gws. This gives the rationale also to revise the assumption of equidistant binning of the breathing phases as well as the selection of the used breathing phases.

When planning the concept of this work, we were aware that we were looking for rather small dosimetric differences. Therefore, we explicitly decided not to use maximum intensity projection (MIP) scans, where the 4DCT image information is compressed into a single CT. Even though, previous works suggest the safety of the use of MIP scans for ITV generation^24^, this might not be applicable for tumors not fully surrounded by lung tissues and in close proximity to other organs such as for central lung tumors^25^. In these cases underestimation of the tumor motion and the ITV might be the consequence that can lead to underdosage of the tumor volume^26,27^. Therefore, the results in this study are based on the 4DCT rather than a MIP reconstruction.

This work reveals the potential for phase gating for SBRT of lung tumors. In particular challenging cases with respect to treatment planning, where the tumor is close to OAR and those that are influenced by a large amount of breathing motion might benefit from the technique presented here. We revealed that the optimal gw size varies on a patient individual basis. In contrast to previously published works, we showed that reduction of the gw size only might not result in the best dosimetric result. Therefore, we suggest a patient individual gw considering tumor position and motion with respect to OAR locations in order to improve the dosimetric results for lung SBRT.

Furthermore, the presented data might question gating as general solution for motion mitigation, especially for central tumors in direct neighborhood of sensitive normal tissue and lung SBRT with ablative doses applied, gating alone might not be able to improve the dose distribution. Therefore, we claim that prediction of dosimetric effectiveness for gating is required on a patient individual basis. In order to reduce laborious contouring using breathing phases and dose simulation, future investigations of the authors focus on a simulation based analyzation method for prediction of gating effectiveness.

## Conclusion

Patient-individual phase gating, considering patient individual tumor position and location, can considerably improve the dose distribution for SBRT of lung tumors. This can be of particular importance for the treatment of central tumors in close proximity to critical organs with high radiation doses.
